# Analysis of Preventive Behaviors of Rural Tourism Hosts in the Face of COVID-19 Pandemic: Application of Health Belief Model

**DOI:** 10.3389/fpubh.2021.793173

**Published:** 2021-12-23

**Authors:** Ali Asghar Mirakzadeh, Faranak Karamian, Ehsan Khosravi, Fatemeh Parvin

**Affiliations:** ^1^Department of Agricultural Extension and Education, Faculty of Agriculture, Razi University, Kermanshah, Iran; ^2^Department of Agriculture Extension and Education, Agricultural College, Razi University, Kermanshah, Iran; ^3^Department of Management and Entrepreneurship, Faculty of Social Sciences, Razi University, Kermanshah, Iran; ^4^Department of Management and Entrepreneurship, Faculty of Social Sciences, Razi University, Kermanshah, Iran

**Keywords:** health perception, rural tourism hosts, health belief model, risk preventative behavior, safety behavior, COVID-19

## Abstract

The novel coronavirus (COVID-19) is one of the most severe public health crises in recent history. Therefore, in order to prevent the spread of COVID-19 and its negative effects on the health of rural tourist hosts and the rural community, it is necessary to pay attention to the conservation and health behaviors of rural tourist hosts. This study was conducted with the purpose of analyzing preventive behaviors of rural tourism hosts in the face of COVID-19 pandemic with the application of the health belief model (HBM) that is one of the most widely used models to study behavior to prevent and control diseases. In this study, all 80 tourism hosts of tourism target villages in Kermanshah province (the west of Iran), were studied as study population. A questionnaire was used to collect data which its validity and reliability were confirmed. Structural equation modeling (SEM) using Smart PLS software was used to analyze the data. The results of SEM indicated that perceived severity, perceived susceptibility, self- efficacy, perceived benefits, and cues to action accounted for 56% of the variance of “COVID-19 preventive health behavior” among the hosts of rural tourists in Kermanshah province. Moreover, the perceived susceptibility was the strongest predictor of preventive health behavior, while perceived barriers were not significant on behavior. Therefore, planning based on the HBM with emphasis on increasing awareness to improve and modify the health behavior of rural tourist hosts is recommended.

## Introduction

The COVID-19 pandemic crisis is a threat to public health for both developing and developed countries (1). The COVID-19 first appeared on December 31, 2019 in Wuhan, China, and then spread rapidly around the world, with the World Health Organization (WHO) declaring it a pandemic crisis on March 11, 2020 (2, 3). Globally, the COVID-19 crisis is seen as a major public health challenge that although not as deadly as the H1N1 flu pandemic, it is unprecedented in terms of the rapid transmission of viral agents from one human to another (4). The COVID-19 pandemic crisis has had devastating effects on various systems such as health, political, social, and especially economic in rural areas (5, 6). In this regard different countries have taken several protective and preventive measures to prevent its spread, which have had the most negative impact on businesses, especially tourism businesses (7, 8).

The tourism industry has long been recognized as one of the most vulnerable sectors to crises (9). Over the past 15 years, many health-related crises, particularly epidemics and pandemics, have severely damaged the tourism industry at the regional, national, and international levels (10). In this regard, the sudden outbreak of the COVID-19 has been an unprecedented shock to the tourism industry (11).

International tourism has declined by more than 80% since the outbreak of the COVID-19 in 2019 (12). The World Tourism Organization (WTO) estimates in 2021 that the outbreak of the COVID-19 will cause $1.3 trillion damage to the tourism industry (13). The emergence of the COVID-19 crisis has also suspended interactions between the origin and destination of tourism in the tourism industry (14) and the unfortunate consequences of the COVID-19 crisis have led to a change in the attitude of the host community, as a result, to their interactions with tourists (15). The tourism host community is aware of health ethics and treatment standards, and seeks to minimize public uncertainty and risk through new behavioral changes (16).

In this regard, Iran was one of the first countries in which the COVID-19 spread rapidly. With the aim of controlling the spread of the COVID-19, the Iranian government has put on the measures such as travel restrictions, social distancing, closure of markets and crowded areas and etc. As a result, the tourism sector was rapidly disrupted, so that these control measures have had a great impact on the country's economy, especially the tourism industry. The prolongation of the COVID-19 crisis and the uncertainty of the end of this crisis and the increase of economic pressure on the government and people of Iran led to the government being forced to reduce restrictions after a few months, and as a result, in mid-March 2020, the outbreak of the COVID-19 was intensified again in Iran (17). In this regard, unfortunately, we have witnessed an intensification of the outbreak of the COVID-19 in Iran during different waves (18). Tourists in Iran were also tired of the continued restrictions and quarantines. Therefore, according to health recommendations, most tourists tended to go to sparsely populated, pristine and natural areas instead of visiting densely populated areas. One of the most important and talented areas is rural areas and the exploitation of rural tourism. The presence of tourists in rural areas during the COVID-19 outbreak, although associated with many opportunities, it has led to the formation of feelings of anxiety, pressure and stress in the life of the rural host community due to the rapid increase in the number of new cases of COVID-19 (19, 20). Currently, government officials, the private sector, and most social researchers are working to develop strategies for planning and redeveloping tourism and building the confidence of tourists to return to their destinations and ensure their health; but an important issue that has been overlooked is the behavior of rural communities hosting tourists during pandemic. Numerous models such as protection motivation theory (21, 22), theory of planned behavior (23, 24), social cognitive theory (25, 26), Johnson's comprehensive model of information seeking (27) have been developed to study behavior (28), which is one of the most popular and widely used models is HBM. Gochman in 1997 defines health behaviors as overt patterns of behavior, practices and habits related to health maintenance, restructuring and improving health (28). The HBM is one of the most important models in the field of health issues that provides strategies to deal with various factors that threaten the health of individuals, such as the COVID-19. This theory was developed by social psychologists, Godfrey Hochbaum and Irwin Rosenstock in the early 1950s (29), to better understand why some people have failed to adopt health prevention programs (30). This theory has provided a useful framework to examine health behaviors and identify key beliefs of health, and has shown acceptable success in predicting a wide range of health behaviors (28). Therefore, in this study, due to the health of tourists and tourism hosts and the validity of the HBM model due to multiple applications in various studies, this model has been used to investigate the health behavior of hosts; this is while other models are less involved in health issues.

Various studies (9, 28, 31–33) show that HBM has had many applications in various topics related to human health behaviors. However, few studies have been conducted using this model during the outbreak of the COVID-19, especially in the field of tourism. For example, although studies such as Mahindarathne (28), and Sreelakshmi and Sangeetha (33) have addressed this model in terms of the conditions created by the COVID-19 outbreak, in both studies the general public has been examined as a statistical population. Also, various studies such as Thams et al. (14), Williams (3), Liew (8), and Abu Bakar and Rosbi (34) emphasized the importance of the tourism industry and the economic effects of the COVID-19 crisis on it. Although Huang et al. (9) used HBM in the field of tourism, this study also focused on tourists and ignores the study of the host community as well as how they react and behave. Therefore, due to the lack of studies as well as the importance of host community behaviors in tourism development, this study uses HBM to investigate the preventive behavior of the tourism host community, which somehow refers to the resilience of tourism hosts in times of crisis to retain their customers.

HBM is one of the most well-known and practical theories of behavioral change and preventative behaviors based on risk perception (35). The outbreak of the COVID-19 has caused fear, anxiety and risk perception in the rural tourism host community. Since the outbreak of the COVID-19 has led to the perception of risk in the rural tourism host community, they believe that the presence of tourists has increased the risk of the COVID-19 in rural communities. Therefore, improve of preventive behaviors in the community is essential. This study seeks to investigate the preventive behaviors of the rural tourism host community against the COVID-19 through HBM in the tourism target villages (more details in the study area section Research Methodology) of Kermanshah province in western Iran. The results of the present study are very important to understand preventive and health behaviors, reduce casualties and increase the safety of the tourist host community, promote health, reduce rural treatment costs, and measure the knowledge and attitude of the host community toward the risks of the COVID-19. Given that rural tourist hosts and also rural population are faced with the threat of the COVID-19 outbreak caused by rural tourists, so what is important is to analyze the preventive behaviors of rural tourist hosts in this situation. In this regard, to provide operational strategies to strengthen preventive behaviors and increase their safety, study of their behavior in this specific condition was the main purpose of this study. In this way, the continuation of the activity of rural businesses and the provision of livelihood for the villagers in the conditions of the COVID-19 crisis will be provided. Specifically, the objectives of this study are: (1) To apply the HBM to explore the preventive behaviors of rural tourist hosts in the face of COVID-19, (2) To identify variables affecting the preventive behavior of the tourism host community, (3) To discuss the appropriateness of the HBM framework and draw conclusions about its predictive power, and (4) To provide operational strategies to promote and improve the host community's preventive behaviors. In general, despite the importance of understanding the behavior of tourism hosts, based on the studies, no HBM study was found to understand the preventive behavior of the tourism host community, especially in rural areas. Therefore, the main purpose of this study is to analyze the preventive behaviors of rural tourism hosts in the face of COVID-19 pandemic with the application of HBM.

## The Health Belief Model

This model focuses on a person's mental perceptions such as perceived susceptibility (Person's opinion about the chance of getting the disease), perceived severity (Person's opinion about the condition, the consequences and complications of the disease), perceived benefits (Person's belief about the usefulness of recommended actions to eliminate the risk or usefulness of accepting a behavior), perceived barriers (Person's perception of the tangible and psychological costs of recommended actions), self-efficacy (Perceived ability of a person to perform an activity) (36, 37) and cues to action (Strategies to activate readiness to take or not to take any actions) (37). Some studies [see (37)] also state that this model is influenced by several factors (moderating factors) such as demographic, socio-cultural, economic, knowledge, and psychological factors.

**Figure 1:**
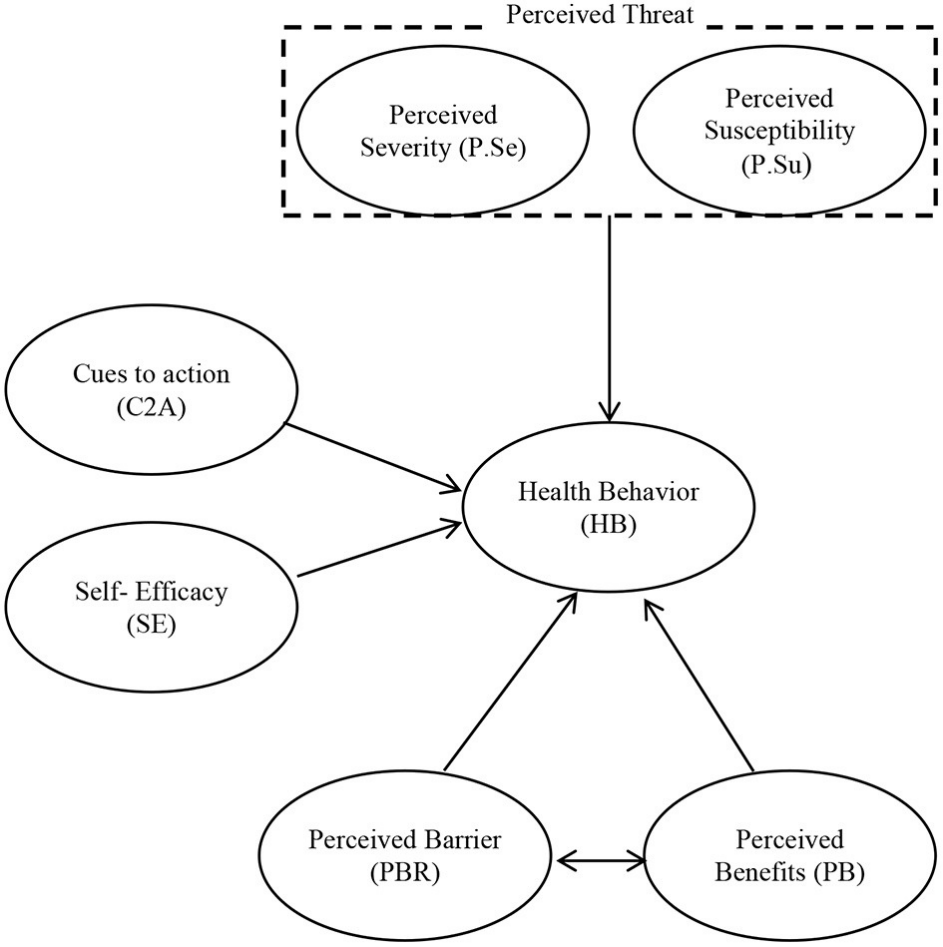
HBM framework (39).

In general, HBM assesses the relationship between health-related beliefs and health preventive behaviors (38). Based on this, it can be said that people's preventive measures depend on their beliefs, because if people feel that they are exposed to susceptibility (perceived susceptibility), they take preventive measures to avoid risks. In other words, if a person believes that a situation is potentially dangerous and can have a significant impact on it (perceived severity) and thinks that he can reduce the risks and complications of the situation by taking a series of measures also, realizing that the benefits of these actions (perceived benefits) outweigh the barriers, the person will be more involved in the required behavior (35). Figure 1 shows the theoretical framework of the HBM.

## Research Methodology

### The Area of Study

**Figure 2:**
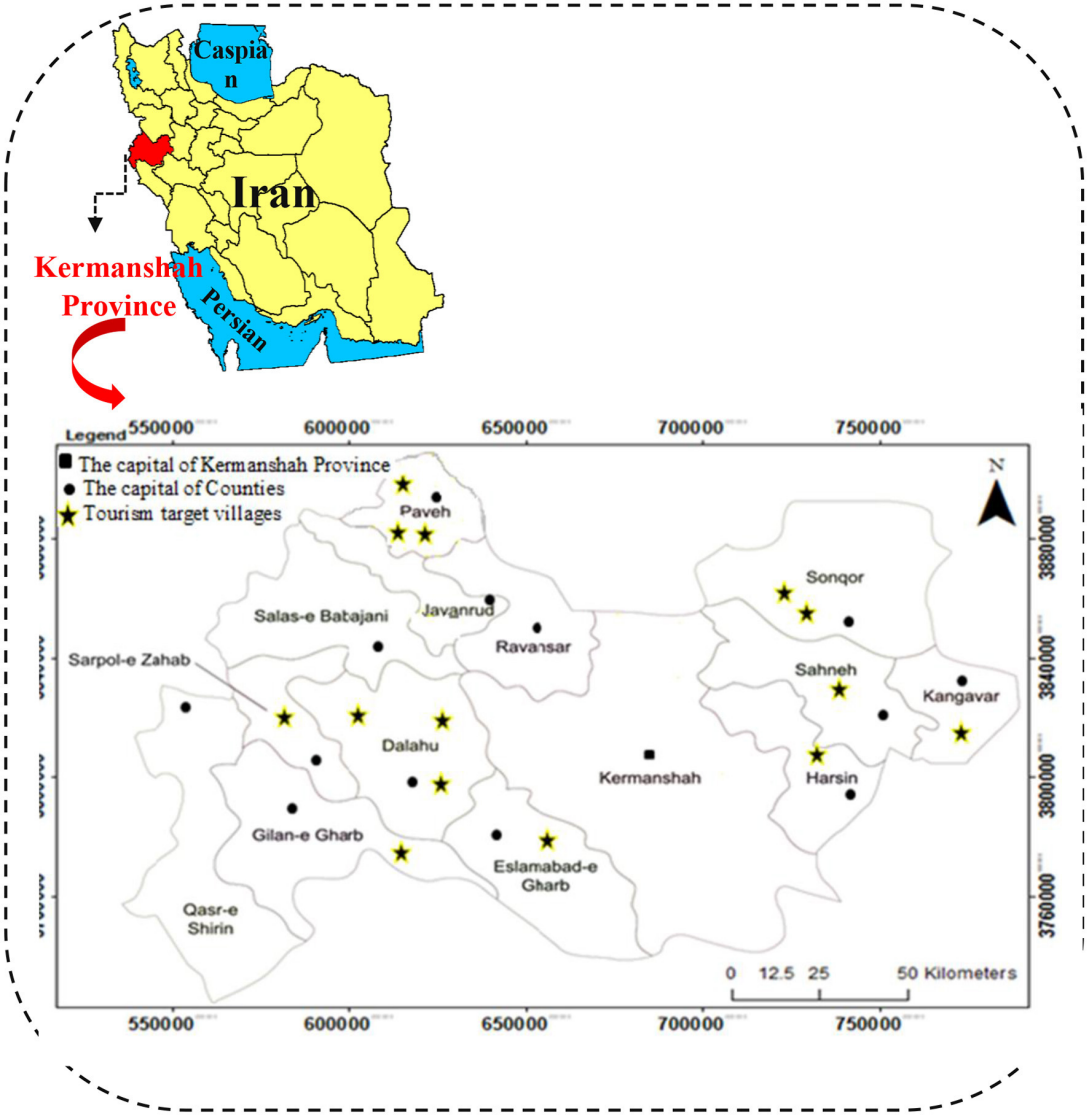
The study area and location of tourist target villages in Kermanshah province.

Kermanshah province is one of the western provinces of Iran and its center is Kermanshah city (Figure 2). The area of Kermanshah province is equal to 24,640 square kilometers (1.5% of the total area of Iran). This province is ranked 17th out of 31 provinces of Iran in terms of area. Kermanshah province is limited to Iraq from the west, Hamedan province from the east, Kurdistan province from the north and Ilam and Lorestan provinces from the south (40). At the time of this research (2020), this province has 14 townships, 31 central parts of the township, 84 districts and 2,793 villages (41). Kermanshah province has a high potential in the field of tourism because this province has pristine nature, suitable climate, four seasons climate, abundant water, numerous border markets, cultural diversity and fertile plains, infrastructure and proper communication routes and international airport. Kermanshah province is one of the oldest provinces of Iran with more than 4,000 known historical and natural monuments, of which more than 2,200 have been nationally registered and works such as Biston and Uramanat region have been registered worldwide (42).

In 2008, 14 villages in Kermanshah province, which are in a much better situation in terms of tourism capacity, were introduced as “tourism target villages”; These villages are: Piran (in Sarpol-e-Zahab township), Harir (in Dalahoo township), Kandoleh (in Sahneh township), Hajij (in Paveh township), Harasam (in Islamabad Gharb township), Shalan (in Dalahoo township), Fash (in Kangavar township), Noji Varan (in Bistoon township), Varmaghan (in Sonqor township), Charmah Olya (in Sonqor township), Khanghah (in Paveh township), Golain (in Gilangharb township), Sorkheh Dizeh (in Dalahoo township), and Shamshir (in Paveh township) (42); The location of the mentioned villages can be seen in Figure 2.

In this province, on February 20, 2020, the first sample of the COVID-19 was identified and officially registered by the Ministry of Health. After that, the COVID-19 spread rapidly throughout the cities of Kermanshah province. Then, the cities of this province have been in the colors of mainly red (very dangerous) and orange (high-risk) colors, and in very few cases in some sparsely populated and low-traffic cities, such as Salas-e Babajani, have yellow (danger) colors. These conditions continued until the writing of the report of this study (i.e., the first of October 2021).

### Study Design and Sample Size

The present study was a descriptive-survey research with a quantitative approach. The present study is a cross-sectional study conducted from September to December 2020 in Kermanshah province in the west of Iran (Figure 2). The statistical population included all rural tourist hosts in 14 tourism target villages in Kermanshah province. Rural tourism hosts meant those villagers who provided accommodation services to rural tourists in rural areas such as rural ecotourism centers. Since the Tourism office of Kermanshah Province provided only the official (formal) number of tourism hosts, so the researchers decided to ask the villagers (Rural officials) of 14 tourism target villages for the number of formally and informally hosts. According to statistics provided by the officials of tourism target villages, 80 tourism hosts are formally and informally providing services to tourists at the time of the survey (2020). Since the number of study populations was small, the census method was used for sampling, so the whole statistical population was selected as a statistical sample (*N* = *n* = 80). Due to the critical situation of the COVID-19 and also the existence of traffic restrictions between cities, a telephone interview was used to complete major questionnaires from September to November 2020. In general, it took about 30–45 min to complete each questionnaire. All tourist hosts were informed that participation in this study was entirely voluntary.

### Study Instrument and Data Collection Process

The data collection tool in this study was a questionnaire whose items and constructs were based on a comprehensive review of previous relevant studies and the theory of health belief (9, 28, 31–33, 35). The questionnaire included closed questions in two general parts: (1) Demographic section and (2) HBM section. In the demographic section, the demographic characteristics of the respondents and characteristics of their business such as age, gender, marital status, level of education, underlying diseases, etc. were examined.

The second part of the questionnaire included items and constructs of HBM that were adjusted according to the COVID-19 crisis based on the experts' opinions. This section included the following constructs: Perceived Susceptibility (P.Su); Perceived Severity (P.Se); Perceived Benefits (PB); Perceived Barriers (PBR); Cues to Action (C2A) [External (C2Ae) and Internal (C2Ai)]; Self-Efficacy (SE); and Health Behavior (HB). All constructs and all items are shown in Table 1.

**Table 1:** The items, constructs, and descriptive results of HBM.

**Construct**		**Item**		**Mean**	**SD**
P.Su		P.Su1	In my opinion, the hosts of rural tourists have a higher risk of the COVID-19 than other occupational groups.	3.54	0.70
		P.Su2	In my opinion, if you do not follow the health protocols, the risk of the COVID-19 increases.		
		P.Su3	I think the more rural tourists we receive, the more likely we are to get the COVID-19.		
		P.Su4	In my opinion, more than 80% of carriers not only do not have obvious symptoms of the COVID-19, but they themselves do not know that they are carriers, and the only way to diagnose it is to do the relevant medical tests.		
		P.Su5	In my opinion, the COVID-19 can be transmitted through various means such as respiration, etc.		
P.Se		P.Se1	I think getting the COVID-19 can affect all aspects of my life.	3.11	0.87
		P.Se2	In my opinion, if I get the COVID-19, I will have high treatment costs.		
		P.Se3	I think getting the COVID-19 can cause irreversible side effects in my body.		
		P.Se4	I think getting the COVID-19 can reduce the vitality in my life.		
		P.Se5	I think getting COVID-19 can be fatal in some cases.		
PB		PB1	In my opinion, the prevention of the COVID-19 reduces the economic costs of disease in the workplace.	3.63	0.72
		PB2	In my opinion, preventive measures against the COVID-19 cause mental health in a person.		
		PB3	In my opinion, the prevention of the COVID-19 reduces its economic costs.		
		PB4	In my opinion, the prevention of the COVID-19 causes the health of my family.		
		PB5	In my opinion, the prevention of the COVID-19, in addition to attracting and retaining tourists provide an opportunity to rebuild infrastructure and address the shortcomings of the service unit.		
PBR		PBR1	In my opinion, the hosts of rural tourists do not have enough time to disinfect the environment due to their busy schedule and excessive fatigue.	4.05	0.61
		PBR2	In my opinion, lack of knowledge about health protocols in the field of the COVID-19 has led to non-compliance with the principles of prevention.		
		PBR3	In my opinion, the unavailability of disinfectants and sanitary facilities such as masks, etc. in rural areas will cause a greater prevalence of the COVID-19.		
		PBR4	In my opinion, psychological problems are one of the effective factors in increasing the incidence of the COVID-19.		
		PBR5	In my opinion, the current economic situation has made it difficult to deal properly with the COVID-19.		
C2A	C2Ae	C2Ae1	In my opinion, the recommendations of your rural health network staff are an incentive to take preventive behaviors.	3.79	0.64
		C2Ae2	In my opinion, the warnings of official authorities such as the WHO, the Ministry of Health of Iran, medical staff and rural health networks are effective in reducing the prevalence of the COVID-19.		
	C2Ai	C2Ai1	In my opinion, observing the condition of patients and their recommendations in the field of the COVID-19 (from acquaintances, relatives or even people who are on TV or virtual networks) can motivate them to take preventive measures.		
		C2Ai2	In my opinion, searching in media such as the Internet and social networks to find ways to prevent the COVID-19 is effective in reducing the prevention.		
		C2Ai3	In my opinion, knowing the daily death rate due to the COVID-19 is effective in performing preventive behaviors.		
		C2Ai4	In my opinion, increasing the awareness of the irreversible side effects of the COVID-19 can be effective in preventive behaviors.		
SE		SE1	In my opinion, despite all the restrictions in the workplace, by learning the principles of prevention and following health protocols, I can prevent coronary heart disease.	3.73	0.70
		SE2	I think I can strengthen my immune system to fight Corona.		
		SE3	In my opinion, despite my busy schedule, I can exercise for at least 30 min daily to strengthen my immune system.		
		SE4	I think I need to improve my diet to strengthen my immune system.		
		SE5	I think I have to respect social distance in the workplace.		
		SE6	I need to use the right mask and gloves at work to prevent the COVID-19.		
		SE7	I think I need to reduce work-related stress in the face of the COVID-19 outbreaks by establishing appropriate communication with rural tourists and other colleagues (virtually or in person with social distance).		
		SE8	I am sure I can prioritize washing and disinfecting my hands and face.		
		SE9	I am sure I can reduce my communication with tourists and others during the day (without harming my business) as much as possible by planning to increase work efficiency.		
HB		HB1	Since the outbreak of the virus, how many people have you suspected of having the COVID-19?	3.60	0.72
		HB2	How much disinfectant and mask have you used since the outbreak of the COVID-19?		
		HB3	Since the outbreak of the COVID-19, to what extent have you minimized social interactions and maintained social distance?		
		HB4	Since the outbreak of the COVID-19, to what extent have you continuously disinfected tourist accommodation?		
		HB5	How much have you exercised to boost your immune system since the outbreak of COVID-19?		
		HB6	Since the outbreak of the COVID-19, to what extent have you controlled your diet to boost your immune system?		
		HB7	Since the outbreak of the COVID-19, to what extent have you followed the correct way to wash your hands and face (hot water and soap for 20–30 s)?		
		HB8	To what extent didn't you leave your home when the quarantine was announced by the government due to the outbreak of the COVID-19 and did you follow the principles of quarantine in hosting tourists?		
		HB9	Since the outbreak of the COVID-19, to what extent have you been constantly following and following up-to-date ways and principles of prevention and health advice from reliable sources?		
		HB10	To what extent have you installed equipment and structural changes (air conditioning, etc.) in your business environment?		
		HB11	To what extent have you been able to raise awareness and share information and experiences about the COVID-19 with other colleagues and locals?		

In the HBM section, all items were measured through a 5-point Likert scale (1 = Strongly Disagree to 5 = Strongly Agree). In order to evaluate the validity of the research tool, content validity and structural validity were used. The content validity of the questionnaire was confirmed by experts in the field of rural tourism and health. Average of variance extracted (AVE) and discriminant validity indexes were used for structural validity. The reliability of the questionnaire constructs was measured through Cronbach's alpha value and composite reliability (CR). Table 1 shows all the items and constructs of the research questionnaire.

### Data Analysis

Data analysis was performed in two sections of descriptive statistics and inferential statistics using SPSS V. 20.0 and Smart PLS software. Structural equation modeling (SEM) was done using Smart PLS software.

## Results

### Descriptive Statistics

#### Demographic Characteristics of Respondents

The results showed that the average age of the respondents was 40 years. Most of the respondents (77.5%) were male. In terms of marital status, 67.5% of the respondents were married. Result showed that in terms of education, 50% of the respondents had diploma and the half of them (50%) had educated in university. The highest percentage of education was related to bachelor's degree with 26.2%. Regarding the underlying diseases (diabetes, kidney disease, cardiovascular disease), 87.5% of the respondents stated that they did not have any diseases. In terms of health education, 66.2% stated that they were educated (including formal or informal education) and 42.5% of the respondents stated that they had received formal health education in order to obtain a health license for their business. Respondents stated that in Kermanshah province, the most prosperous seasons for tourism businesses are spring, summer and winter. Eighty percentage of the respondents stated that the number of tourists has decreased after the outbreak of the COVID-19.

#### Descriptive Analysis of Constructs and Items

The items, constructs, and descriptive results of HBM constructs are shown in Table 1. As Table 1 shows, the average score of all constructs in HBM is higher than 3 (out of 5). Based on the results, perceived barrier had the highest mean score among hosts (Mean = 4.05 out of 5, SD = 0.61). After that, cues to action had a mean of 3.79 out of 5 (SD = 0.64). Perceived severity had the lowest mean score (Mean = 3.11 out of 5, SD = 0.87). In addition, the results of a descriptive analysis of the health behavior of rural tourism hosts against the COVID-19 showed that they have a high preventive behavior (Mean = 3.60 out of 5, SD = 0.72) (Table 1).

### Results of SEM Analysis

First, the fit and validity of the measurement model were verified through confirmatory factor analysis (CFA). Six items with standardized factor loadings of 0.5 or less were removed. In order to evaluate the model fit, 5 indices including the following were used: SRMR < 0.10, D_G > 0.05, D_LS > 0.05, NFI > 0.90, RMS_ Theta ≤ 0.12 (43). In this study, after model saturation all model fit indices were higher than the recommended value. Therefore, the research model had an appropriate fit (Table 2). Table 3 shows the values of AVE and CR. The recommended values are 0.5 and 0.6, respectively (43). Table 3 also shows the diagnostic validity. The AVE for the research constructs (0.726 < AVE < 0.921) was greater than their correlation (0.005 < r < 0.657), which indicated that the diagnostic validity of the structures in the proposed research model was confirmed. So, the validity and reliability of all latent variables in the proposed model are at an acceptable level.

**Table 2:** Fit indices for the measurement model.

**Fit index**	**SRMR**	**D_G**	**D_LS**	**NFI**	**RMS-Theta**
Recommended value	<0.10	>0.05	>0.05	>0.90	≤ 0.12
Estimated value	0.09	3.05	5.87	0.91	0.11

**Table 3:** Diagnostic validity, AVE, and CR.

**Constructs**	**Diagnostic validity**							**AVE**	**CR**
	**C2A**	**HB**	**P.Se**	**PB**	**P.Su**	**PBR**	**SE**		
C2A	0.921^a^							0.848	0.918
HB	0.385^b^	0.741^a^						0.550	0.930
P.Se	0.122^b^	0.444^b^	0.755^a^					0.570	0.868
PB	0.177^b^	0.623^b^	0.413^b^	0.726^a^				0.527	0.848
P.Su	0.500^b^	0.657^b^	0.318^b^	0.501^b^	0.739^a^			0.547	0.856
PBR	0.054^b^	−0.097^b^	−0.056^b^	−0.005^b^	0.027^b^	0.785^a^		0.617	0.824
SE	0.602^b^	0.523^b^	0.206^b^	0.441^b^	0.648^b^	0.167^b^	0.816^a^	0.666	0.909

a *The square roots of AVE estimate*.

b *Correlation is significant at the < 0.01 level*.

**Figure 3:**
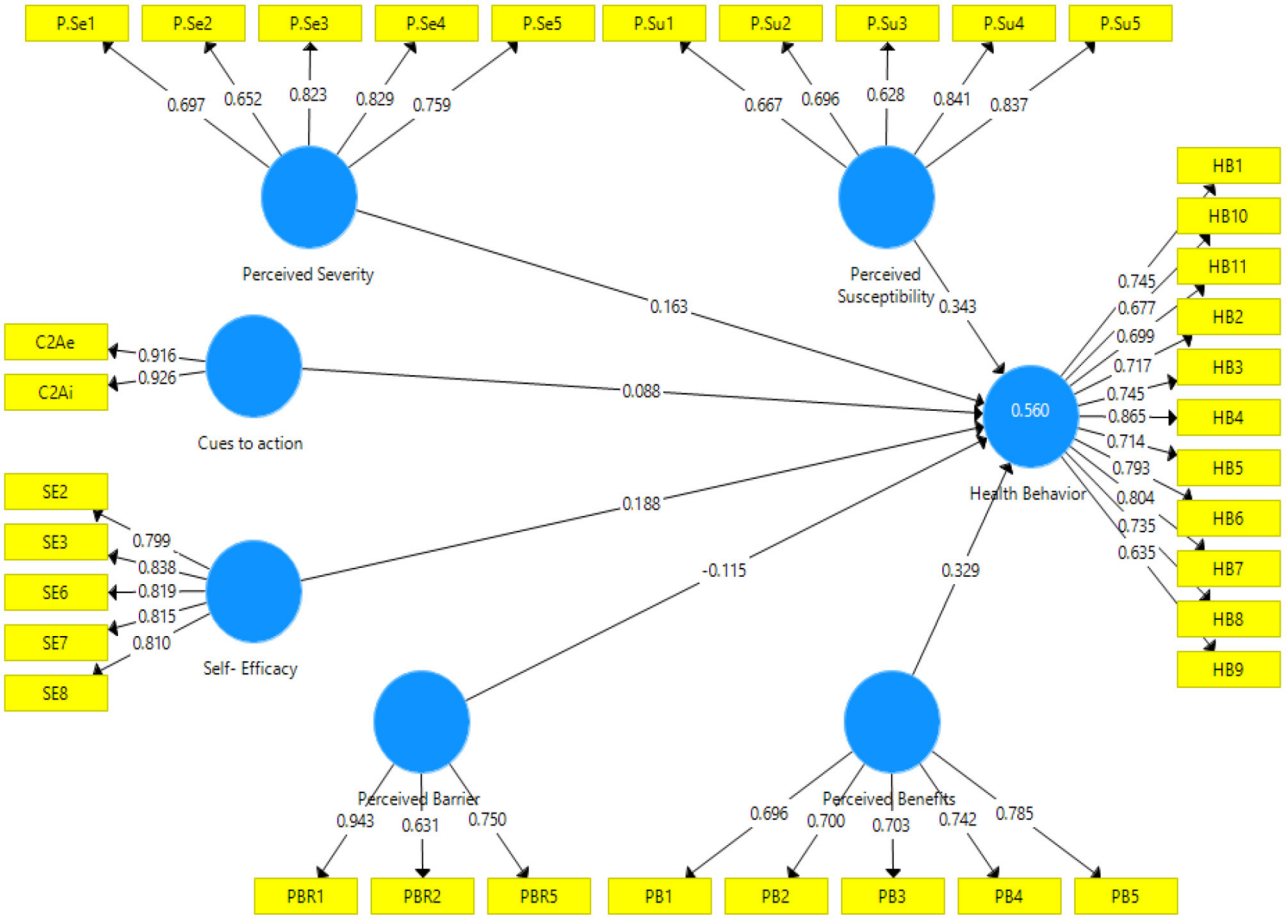
Structural path saturated model.

SEM analysis showed that perceived severity, perceived susceptibility, self- efficacy, perceived benefits, and cues to action could predict 56% of the variance of “COVID-19 preventive health behavior” among the hosts of rural tourists in Kermanshah province. Also, according to the results, perceived barrier did not have a significant effect on preventive behavior. The results showed that the most important predictor of COVID-19 prevention behavior is perceived susceptibility (β = 0.34, *P* < 0.00). In this regard, cues to action has the least power in predicting the health behavior of rural tourism hosts against COVID-19 (β = 0.09, *P* < 0.00) (Figure 3). Table 4 shows Cronbach's alpha, standardized factor loadings, *t*-value value and standardized beta coefficients in saturated model.

**Table 4:** Cronbach's alpha, beta coefficients, *t*-value and factor loadings in saturated model.

**Constructs and items**	**Factor loading**	***t*-value**
PB (α = 0.79) (β = 0.33^**^)		
PB1	0.69	7.48
PB2	0.70	7.58
PB3	0.70	6.25
PB4	0.74	8.57
PB5	0.78	11.44
P.Se (α = 0.82) (β = 0.16^*^)		
P.Se1	0.69	8.96
P.Se2	0.65	7.99
P.Se3	0.82	10.63
P.Se4	0.83	12.20
P.Se5	0.75	7.24
P.Su (α = 0.77) (β = 0.34^**^)		
P.Su1	0.67	8.09
P.Su2	0.70	9.16
P.Su3	0.63	5.60
P.Su4	0.84	21.36
P.Su5	0.84	23.28
**PBR (α = 0.71) (β = −** **0.11^ns^)**		
PBR1	0.94	2.90
PBR2	0.63	1.99
PBR5	0.75	2.26
C2A (α = 0.82) (β = 0.09^**^)		
C2Ae (It consisted of two items that were computed for the model)	0.91	14.89
C2Ai (It consisted of four items that were computed for the model)	0.92	9.33
SE (α = 0.88) (β = 0.18^**^)		
SE2	0.80	15.16
SE3	0.83	25.17
SE6	0.82	16.92
SE7	0.81	17.29
SE8	0.81	20.91
**Health behavior (HB) (α = 0.91) (**$R_{Behavior}^{2}$ **= 0.56)**		
HB1	0.74	12.22
HB2	0.72	11.01
HB3	0.74	11.95
HB4	0.86	30.62
HB5	0.71	10.36
HB6	0.79	20.22
HB7	0.80	20.97
HB8	0.73	14.27
HB9	0.63	7.24
HB10	0.68	9.82
HB11	0.70	8.93

* *P < 0.05*.

** *P < 0.01*.

ns *Non-significant*.

## Discussion

The main purpose of this study was to investigate the predictors of preventive behaviors of rural tourism hosts in the face of COVID-19 crisis, which in this regard, the HBM model was used as a theoretical model. The results of SEM indicated that perceived severity, perceived susceptibility, self- efficacy, perceived benefits, and cues to action accounted for 56% of the variance of “COVID-19 preventive health behavior” among the hosts of rural tourists in Kermanshah province. Moreover, the perceived susceptibility was the strongest predictor of preventive health behavior, while perceived barriers did not have a significant impact on behavior. Therefore, the HBM is a good model to explain and predict preventive rural tourist hosts' behaviors in face of COVID-19 pandemic. This finding is important because the study was conducted in the context of the COVID-19 crisis. Because despite the frequent changes in policies and programs, as well as the lack of public confidence in health advice, the changing behavior of the COVID-19 pandemic, the spread of rumors in the political space and uncertainty about the future, this model has been able to provide relatively valuable guidance to researchers and executives in dealing with such crises. While this model has been used in previous studies [see (35)] under normal conditions and in various subjects, that are not comparable to COVID-19, and in comparison with the recent studies that have used this model in the context of the COVID-19 crisis [see (28)], the findings are significant.

Perceived susceptibility of COVID-19 prevention was the most powerful predictor of preventive health behavior, which is consistent with the studies conducted by Alhazmi et al. (32), Sreelakshmi and Sangeetha (33), Tajeri moghadam et al. (35), and Huang et al. (9). In this regard, it can be said that the nature of tourism in dealing with contagious diseases such as COVID-19 due to the possibility of getting infected and the transmission of the virus by tourists coming from different places, makes tourism hosts more sensitive to this issue. Another reason for this is that the families of tourism hosts are at risk. In this regard, the HBM model has shown its ability to highlight the effect of this variable on behavior. In addition, this finding indicates that rural tourism hosts have realized the susceptibility of the situation and this makes them ready and resilient in the face of such crises. According to this, if tourism hosts accept that they are susceptible to the disease and there is a possibility of infection and consequent harm to them, then they are more likely to follow preventive behaviors, and through this perceived susceptibility, they will predict appropriate health behavior. Therefore, updating the knowledge of tourism hosts through valid and timely awareness can be considered as a key factor in the management of COVID-19 crisis in the rural tourism industry.

Given the expansion of virtual networks and the elimination of time and distance, networking is a possible measure in crisis management in rural communities in the study area. Also, since the pandemic conditions are the same for all the villages of the province and the country, the possibility of applying the results of this research in other parts of the country depends on environmental conditions, especially the timely action of managers and officials in decision making and informing stakeholders. The results showed that perceived severity also has a positive and significant effect on the incidence of health behavior. This finding is in accordance with the studies of Alhazmi et al. (32), and Sreelakshmi and Sangeetha (33), but it is not similar to results of study Tajeri moghadam et al. (35). This finding shows that the tourists' hosts have assessed COVID-19 as a serious and dangerous disease for their health as well as their business, which can have serious consequences in rural health, and therefore on various dimensions such as economic, social, cultural and political situation of rural areas. Therefore, perceived severity can lead to preventive behaviors. Since the COVID-19 disease has never been similar in the study area and also, during the outbreak of the disease, the hosts of rural tourism were in quarantine, the effect of perceived severity is lower than other factors and it only has more predictive power than cues to action. Based on this finding, institutions and trustees of rural tourism businesses and community health can reveal the facts of the crisis by sharing the experiences, pathology of behaviors and actions of rural tourism activists and reduce the probability of misunderstanding of the severity of the crisis and lack of proper and timely behavior by tourism hosts. Since time is of the essence in such cases, perceived intensity can have a significant effect on reducing the effects of the crisis, and the results of this study also show the positive effect of perceived intensity on the behavior of actors.

The results showed that the effect of perceived benefits of protecting behaviors by hosts had a significant effect on the type of action they took in the face of COVID-19. This result is parallel with studies of Mahindarathne (28), Tajeri moghadam et al. (35), and Huang et al. (9). One of the reasons that can justify this positive effect is the susceptibility of the tourism business to COVID-19. Accordingly, the hosts have made every effort to maintain the job and attract customers by carrying out protective behaviors and have understood its benefits. This has had a positive effect on the continuation of preventive behaviors. Therefore, since the experience of COVID-19 has had a positive effect on improving the health behavior of hosts, it is suggested that by gathering the lived experience of the hosts and compiling it in the form of local tourism health protocols, these protocols be transferred to other similar occupations through the training program. In this way, the readiness and resilience of tourism hosts and rural services can be increased in cases similar to COVID-19.

Also the results showed that perceived barriers did not have a significant effect on health behavior. The insignificance of perceived barriers to host protection behavior may be due to the fact that the motivation to control and manage the effects of COVID-19 on tourism businesses and deleting its negative effects was so great that the existing barriers to preventive behaviors were not understood or did not matter to the hosts. This result is parallel with the study of Tajeri moghadam et al. (35), but it is not similar to the results of Mahindarathne (28), which showed perceived barriers had ability to predict behavior. This may be due to the different nature of the subject of COVID-19 with the subjects pursued in the mentioned research. One reason for this result in comparison to other research results (28) may be the place and sample of the study. Also this may be due to the different nature of the subject of COVID-19 with the subjects that have been studied in previous research.

The results showed that cues to action have a positive and significant effect on health behavior, which is consistent with the study conducted by Tajeri moghadam et al. (35). In other words, recommendations and warnings at the macro and micro levels have led to preventive behaviors. In this study, warnings at the macro level of society such as the WHO, the Ministry of Health of Iran and social channels, as well as at the micro level, the recommendations of regional health experts and other local social networks in the region and villages as cues to action were emphasized. Overall, the results showed that cues to action have a positive and significant effect on health behaviors, but its predictive power is the lowest among the other studied constructs. This shows that if we consider the haste and multiple and sometimes contradictory decisions of officials at the macro level regarding the management of COVID-19, the study model has shown the effect of the variable of “cues to action” at the lowest level compared to other structures of the model. It seems that due to the fact that most of these businesses are located in pristine and less developed areas and these areas are not in a good position in terms of communication infrastructure, cues to action in practice does not have high power in predicting preventive behaviors. Therefore, in this regard, it is recommended that the information communication infrastructure of these areas be upgraded to increase the hosts' access to the most up-to-date information. In this way, the incidence of preventive behaviors in accordance with the most up-to-date health protocols will increase. It is also recommended that in times of crisis, a group of career counselors and health counselors provide expert advice at the appropriate time in order to take timely action by hosts to mitigate the effects of the crisis.

The results showed that self-efficacy has a positive and significant effect on health behavior. This finding is in accordance with the studies of Mahindarathne (28), Huang et al. (9), and Sreelakshmi and Sangeetha (33). The coefficient of this variable indicates that tourism hosts have a good understanding of Susceptibility in the face of COVID-19 and have had considerable self-confidence and self-efficacy when confronted with it, but the predictive power of the “cues to action” variable (which can most likely be related to the haste of officials and the frequent changes in decisions and programs and sensitive conditions governing the society) is very low for various reasons. In other words, in the face of the COVID-19 crisis, what goes back to the individual and to the area of individual decisions and authority has a more positive effect on behavior, than variables related to environmental conditions (e.g., decisions of managers and officials and informing systems).

## Conclusion

This research was conducted due to the COVID-19 crisis in the world and its negative effects on various communities, especially rural communities. In rural communities, non-agricultural occupation has an undeniable effect on the sustainability of rural livelihoods. One of these jobs is rural tourism, which has been severely affected by COVID-19. Hence, the sustainability of these jobs and the livelihood of this group of rural actors depend on the behavior of managers and hosts of rural tourists to manage the risks posed by COVID-19. Therefore, this study has used the HBM model to study the behavior of this community in the COVID-19 crisis to determine whether this model can be used in critical situations to manage behavior in rural communities with its own unique complexities. The results of the research are important both in practical and scientific dimensions. In the scientific dimension, the application of HBM model in analyzing the behavior of rural tourist hosts (rural community) in times of crisis is possible and justifiable. The importance of this finding in this dimension is that so far the behavior of the host community and tourism managers has not been addressed in previous studies and in most of them the behavior of tourists has been considered. In the executive dimension, the results of the research showed that despite the surprise of the COVID-19 pandemic, rural tourism hosts have increasingly relied on individual capabilities such as self-efficacy, understanding the susceptibility and severity of the event, as well as the benefits of their protective behaviors to manage the effects of the disease, and what has been related to the conditions of environment, especially the political and economic environment that has been less able to influence hosts to provide preventive behaviors. As in this regard, the effect of cues to action on protective behavior has been negligible. In other words, in the absence of educational and support services, this society has been able to manage its business with the behavior resulting from the understanding of the situation. Therefore, it is suggested that the trustees, by recording the lived experience of rural actors (at the national and international level) in the face of the COVID-19 crisis and similar cases, take action to realize (community-based) behavioral protocols in national and international level.

## Limitations and Future Research Directions

The most important limitations of this research include the following: This study was performed in the context of COVID-19, which resulted in non-cooperation of some samples due to the fear of COVID-19 infection; due to traffic restrictions in Kermanshah province and the prevalence of COVID-19, physical presence in tourist centers in the villages was difficult; having access to the hosts of rural tourists was very time consuming and costly due to the dispersion of villages in Kermanshah province; and due to the low literacy of some rural tourist hosts, completing some of the questionnaires took time. Also, in order to conduct future research, a comparison of the planned behavior model and the health behavior model in terms of the power of predicting health behaviors among the hosts of rural tourists is proposed.

## Data Availability Statement

The original contributions presented in the study are included in the article/supplementary material, further inquiries can be directed to the corresponding author/s.

## Author Contributions

AAM, FK, and EK contributed to conception and design of the study and performed the statistical analysis. FK and EK wrote the first draft of the manuscript. FP contributed in data collection. AAM, FK, and EK wrote sections of the manuscript. All authors contributed to manuscript revision, read, and approved the submitted version.

## Conflict of Interest

The authors declare that the research was conducted in the absence of any commercial or financial relationships that could be construed as a potential conflict of interest.

## Publisher's Note

All claims expressed in this article are solely those of the authors and do not necessarily represent those of their affiliated organizations, or those of the publisher, the editors and the reviewers. Any product that may be evaluated in this article, or claim that may be made by its manufacturer, is not guaranteed or endorsed by the publisher.

## References

[1] Sarwar F Panatik SA Sarwar F. Editorial: psychology of preventive behavior for COVID-19 outbreak. J Res Psychol. (2020) 2:1–3. 10.31580/jrp.v2i1.1370

[2] Khalid U Okafo LE Burzynska K. Does the size of the tourism sector influence the economic policy response to the COVID-19 pandemic? Curr Iss Tour. (2021) 24:2801–20. 10.1080/13683500.2021.1874311

[3] Williams CC. Impacts of the coronavirus pandemic on Europe's tourism industry: addressing tourism enterprises and workers in the undeclared economy. Int J Tour Res. (2020) 23:79–88. 10.1002/jtr.239525855820

[4] Yazdanpanah M Tajeri Moghadam M Savari M Zobeidi T Sieber S Löhr K. The impact of livelihood assets on the food security of farmers in Southern Iran during the COVID-19 pandemic. Int J Environ Res Public Health. (2021) 18:1–18. 10.3390/ijerph18105310 34067638 PMC8156269

[5] Fuchs C. Everyday life and everyday communication in coronavirus capitalism. TripleC Commun Capital Critiq J Glob Sustain Inform Soc. (2020) 18:375–99. 10.31269/triplec.v18i1.1167

[6] Gretzel U Fuchs M Baggio R Hoepken W Law R Neidhardt J. e-Tourism beyond COVID-19: a call for transformative research. Inform Technol Tour. (2020) 22:187–203. 10.1007/s40558-020-00181-3

[7] Bhaskara GI Viachaslau F. The COVID-19 pandemic and organisational learning for disaster planning and management: a perspective of tourism businesses from a destination prone to consecutive disasters. J Hospital Tour Manage. (2021) 1:364–75. 10.1016/j.jhtm.2021.01.011

[8] Liew VKS. The effect of novel coronavirus pandemic on tourism share prices. J Tour Futures. (2020) 6:1–16. 10.1108/JTF-03-2020-0045

[9] Huang X Dai S Xu H. Predicting tourists' health risk preventative behavior and travelling satisfaction in Tibet: combining the theory of planned behavior and health belief model. Tour Manage Perspect. (2020) 33:100589. 10.1016/j.tmp.2019.100589

[10] Yu M Li Z Yu Z He J Zhou J. Communication related health crisis on social media: a case of COVID-19 outbreak. Curr Iss Tour. (2020) 24:2699–705. 10.1080/13683500.2020.1752632

[11] Yang F Wong IA. The social crisis after-math: tourist well-being during the COVID-19 outbreak. J Sustain Tour. (2021) 29:859–78. 10.1080/09669582.2020.1843047

[12] Luo JM Lam CF. Travel anxiety, risk atti-tude and travel intentions towards “travel bubble” destinations in Hong Kong: effect of the fear of COVID-19. Int J Environ Res Public Health. (2020) 17:7859. 10.3390/ijerph17217859 33120949 PMC7672589

[13] Rokni L. The psychological consequences of COVID-19 pandemic in tourism sector: a systematic review. Iran J Public Health. (2021) 50:1743–56. 10.18502/ijph.v50i9.7045 34722369 PMC8542819

[14] Thams A Zech N Rempel D Ayia-Koi A. An initial assessment of economic impacts and operational challenges for the tourism & hospitality industry due to COVID-19. IUBH Discussion Papers Tour Hosp. (2020) 2:1–16. http://hdl.handle.net/10419/216762

[15] Gössling S Scott D Hall CM. Pandemics, tourism and global change: a rapid assessment of COVID-19. J Sustain Tour. (2020) 29:1–20. 10.1080/09669582.2020.1758708

[16] Lapointe D. Reconnecting tourism after COVID-19: the paradox of alterity in tourism areas. Tour Geogr. (2020) 22:633–8. 10.1080/14616688.2020.1762115

[17] Mirzaei R Sadin M Pedram M. Tourism and COVID-19: changes in travel patterns and tourists' behavior in Iran. J Tour Futures. (2021) 1–13. 10.1108/JTF-01-2021-0017

[18] Ministry of Health of Iran. Corona Virus News. Available online at: https://behdasht.gov.ir (In persian) (2021). (accessed September 15, 2021).

[19] Zhang Y Ma ZF. Impact of the COVID-19 pandemic on mental health and quality of life among local residents in Liaoning Province, China: a cross-sectional study. Int J Environ Res Public Health. (2020) 17:2381. 10.3390/ijerph17072381 32244498 PMC7177660

[20] Higgins-Desbiolles F. Socialising tourism for social and ecological justice after COVID-19. Tour Geogr. (2020) 23:610–23. 10.1080/14616688.2020.1757748

[21] Westcott R Ronan K Bambrick H Taylor M. Expanding protection motivation theory: investigating an application to animal owners and emergency responders in bushfire emergencies. BMC Psychol. (2017) 5:1–14. 10.1186/s40359-017-0182-3 28446229 PMC5406887

[22] Oakley M Himmelweit SM Leinster P Casado MR. Protection motivation theory: a proposed theoretical extension and moving beyond rationality—the case of flooding. Water. (2020) 12:1–14. 10.3390/w12071848

[23] Ulker-Demirel E Ciftci G. A systematic literature review of the theory of planned behavior in tourism, leisure and hospitality management research. J Hosp Tour Manage. (2020) 43:209–19. 10.1016/j.jhtm.2020.04.003

[24] Yazdanpanah M Forouzani M. Application of the theory of planned behaviour to predict Iranian students' intention to purchase organic food. J Clean Prod. (2015) 107:342–52. 10.1016/j.jclepro.2015.02.071

[25] Bandura A. Self-efficacy mechanism in human agency. Am Psychol. (1982) 37:122–47. 10.1037/0003-066X.37.2.122

[26] Yazdanpanah M Zobeidi T Tajeri Moghadam M Nadejda K Katharina L Stefan S. Cognitive theory of stress and farmers' responses to the COVID 19 shock; a model to assess coping behaviors with stress among farmers in southern Iran. Int J Disast Risk Reduct. (2021) 64:102513. 10.1016/j.ijdrr.2021.102513 PMC9766011 36570385

[27] Johnson CM Johnson TR Zhang JA. User-centered framework for redesigning health care interfaces. J Biomed Inform. (2005) 38:75–87. 10.1016/j.jbi.2004.11.005 15694887

[28] Mahindarathne PP. Assessing COVID-19 preventive behaviours using the health belief model: a Sri Lankan study. J Taibah Univ Med Sci. (2021) 16:1–6. 10.1016/j.jtumed.2021.07.006 34393699 PMC8353659

[29] Strecher VJ Rosenstock IM. The health belief model. Cambridge Handbook of Psychology. Health Med. (1997) 113–117.

[30] Bakhtiyari Z Yazdanpanah M Forouzani M Kazemi N. Intention of agricultural professionals toward biofuels in Iran: implications for energy security, society, and policy. Renew Sustain Energy Rev. (2017) 69:341–9. 10.1016/j.rser.2016.11.165

[31] Vaezipour Z Gharlipour Z Mohebi S Sharifirad G. Effect of education on promoting preventive behaviors of oral and dental problems: applying health belief model. Health Educ Health Promot. (2018) 6:135–41. 10.29252/HEHP.6.4.135

[32] Alhazmi AS Al Agili DE Aldossary MS Hakami SM Almalki BY Alkhaldi AS. Factors associated with the use of fashion braces of the Saudi Arabian Youth: application of the Health Belief Model. BMC Oral Health. (2021) 21:1–9. 10.1186/s12903-021-01609-w 33971859 PMC8108328

[33] Sreelakshmi CC Sangeetha KP. Continuance adoption of mobilebased payments in Covid-19 context: an integrated framework of health belief model and expectation confirmation model. Int J Pervasive Comput Commun. (2020) 16:351–69. 10.1108/IJPCC-06-2020-0069

[34] Abu Bakar N Rosbi S. Effect of Coronavirus disease (COVID-19) to tourism industry. Int J Adv Eng Res Sci. (2020) 7:189–93. 10.22161/ijaers.74.23

[35] Tajeri moghadam M Raheli H Zarifian S Yazdanpanah M. The power of the health belief model (HBM) to predict water demand management: a case study of farmers' water conservation in Iran. J Environ Manage. (2020) 263:110388. 10.1016/j.jenvman.2020.110388 32174529

[36] Yue Z Weilin C Li Q Bin W. Patient education and counseling application of the health belief model to improve the understanding of antihypertensive medication adherence among Chinese patients. Patient Educ Couns. (2015) 98:669–73. 10.1016/j.pec.2015.02.007 25746128

[37] Rosenstock IM. Historical origins of the health belief model. Health Educ Behav. (1974) 2:328–35. 10.1177/10901981740020040312859788

[38] Razmara A Aghamolaei T Madani A Hosseini Z Zare S. Prediction of safe driving behaviours based on health belief model: the case of taxi drivers in Bandar Abbas, Iran. BMC Public Health. (2018) 18:1–8. 10.1186/s12889-018-5300-5 29558924 PMC5859486

[39] Rosenstock IM. The health belief model and preventive health behavior. Health Educ Behav. (1974) 2:354–86. 10.1177/109019817400200405

[40] Naderi N Khosravi E Azadi H Karamian F Viira AH Nadiri H. Barriers to developing social entrepreneurship in NGOs: application of grounded theory in Western Iran. J Soc Entrepreneurship. (2020) 12:1–23. 10.1080/19420676.2020.1765409

[41] Management and Planning Organization of Kermanshah Province. Statistical Yearbooks of Kermanshah Province. (2021). Available online at: https://www.amar.mpo-ksh.ir (In Persian) (accessed September 10, 2021).

[42] Tourism office of Kermanshah Province (2021). Statistics of the number of antiquities registered in Kermanshah province. Available online at: https://kermanshah.mcth.ir (In Persian).

[43] Ahmadi P Rahimian M Ghanbari Movahed R. Theory of planned behavior to predict consumer behavior in using products irrigated with purified wastewater in Iran consumer. J Clean Prod. (2021) 296:126359. 10.1016/j.jclepro.2021.126359

